# The Effects of Waterborne Polyurethane-Modified Cement on the Mechanical Characterization of Grouting Concretion Stone

**DOI:** 10.3390/ma17235720

**Published:** 2024-11-22

**Authors:** Jingyu Zhang, Sili Chen, Xinchao Duan, Jinzhu Meng, Junxiang Wang

**Affiliations:** 1 School of Materials Science and Engineering, Shenyang University of Technology, Shenyang 110870, China; zjy7019@sut.edu.cn; 2 School of Architecture and Civil Engineering, Shenyang University of Technology, Shenyang 110870, China; xinchaoduan11111@163.com (X.D.); jinzhumeng@sut.edu.cn (J.M.); wangjunxiang@sut.edu.cn (J.W.)

**Keywords:** waterborne polyurethane (WPU), hydroxypropyl methyl cellulose (HPMC), water-reducing agent (WRA), mechanical properties, grouting concretion stone, energy analysis

## Abstract

To improve the safety and stability of tunnel structures, developing grouting materials suitable for cold regions with excellent performance is crucial. Herein, waterborne polyurethane (WPU) was used to modify cement grouting materials. Through orthogonal testing analysis, the optimal mixing ratio of the modified cement grouting materials was determined to be as follows: a water–cement ratio of 0.5, hydroxypropyl methyl cellulose (HPMC) content of 0.05%, WPU content of 5%, water-reducing agent (WRA) content of 0.2%. Furthermore, the dynamic mechanical properties of grouting concretion stones were studied. The influence of various external parameters on the compressive strength of the grouting concretion stones cured for different ages was evaluated. The influence degree of stone particle size on the dynamic compressive strength of the grouting stone body was d_5–10 mm_ > d_5–20 mm_ > d_5–30 mm_. The split Hopkinson pressure bar experiment was performed to show that for the same strain rate, the absorbed energy and energy utilization rate first increase and then decrease with increasing stone particle size. When the stone particle size was 5–20 mm, the absorption energy and energy utilization rate of the grouting stone body were the highest.

## 1. Introduction

The development of global infrastructure construction poses the challenge of intricate geological conditions [1], and various types of geologic engineering hazards frequently occur [2]. Subway tunnels are usually shallow and often pass through water-rich sand layers. In the construction of subway tunnels, water leakage treatment has become a great challenge. Rock bodies are susceptible to cyclic loading from railroads [3] due to their high contents of water and degree of fragmentation [4], as well as the special characteristics of cold environments [5], which cause rock body instability during engineering excavation and easily induce groundwater phenomena [6]. In the face of such disasters, grouting is a widely recognized effective solution [7]. Grouting can fill broken rock bodies with water-bearing fissures, reduce the water-richness of the stratum, and improve the water-insulating thickness of surrounding rocks, thereby preventing the occurrence of engineering geologic disasters. Permeation grouting with cement-based grout has become an effective method for water leakage treatment because of its convenient construction and friendliness to the environment. Permeation grouting is an effective method used for enhancing stabilization and reducing the permeability of granular soil deposits without disturbing the original soil structure. However, during grouting, whether the performance of the grouting materials can meet the engineering requirements is critical. Compared with other commonly used grouting materials, waterborne polyurethane (WPU) materials have good adhesion, excellent elasticity, and durability. WPU can effectively resist the corrosion of ions in groundwater [8], as well as the influence of freeze–thaw cycles [9], thereby guaranteeing the long-term stability and reliability of the reinforcement effect. The nodular body formed after grouting not only needs to withstand static loads but also may face dynamic effects, such as blast impact and drilling vibration [10], and these dynamic loads impose higher requirements on the dynamic mechanical properties of the nodular body [11]. Therefore, evaluating the performance of grouting concretion stones under dynamic loading, especially their dynamic mechanical properties, is crucial to ensuring the safety and overall stability of projects [12].

Polyurethane (PU) materials are synthetic resins with diverse product forms [13] and multifunctionality, widely used in the transportation, construction, machinery, national defense, sports, and healthcare fields [14]. Fan et al. [15] formulated waterborne aliphatic PU-modified concrete (WPMC) using waterborne aliphatic PU, cementitious materials, coarse and fine aggregates, polycarboxylate superplasticizers, and foam stabilizers. The experimental results showed that an appropriate addition of WPU could increase the modulus of elasticity, compressive strength, split tensile strength, and flexural strength of WPMC. Okumura et al. [16] reported that the bond performance of polymer concrete and cement concrete was closely related to the interface roughness and the interfacial agents, and the bond and tensile strengths of the WPU-modified materials at the age of 28 d were 13 and 6 MPa, respectively. Zhang et al. [17] prepared 11 groups of PU-modified cement concrete specimens with different contents of basalt aggregate, and Li et al. [18] researched rubber powder and polyethylene fiber materials and used split Hopkinson pressure bars (SHPB) to test the specimens. Laqsum et al. [19] reported the results showed that the compressive strength of concrete containing PU binder only revealed a significant decrease with increased PU binder content. Ran et al. [20] used cement, red mud, fly ash, PU, and other materials to modify cement-based grouting materials, studied the grouting mechanism of the modified grouting materials in asphalt pavement settlement, and reported that there was no obvious misalignment effect after grouting via probing radar mapping, indicating that the effect of grouting was strong.

The effect of WPU addition on the working performance of cement-based grouting materials has attracted considerable attention. Pillai et al. [21] studied how the performance of these materials in areas such as compressive strength, split tensile strength, resistance to thermal exposure, abrasion resistance performed. Compared to cement and silica fume-incorporated mortar, the mixes modified with PU-nano silica shows enhancement in split tensile strength. Ruobing [22] used solvent-free self-emulsifying WPU to modify cement; the results showed that increasing the WPU content decreases the slurry fluidity and prolongs the setting time. Al-kahtani et al. [23] investigated the effect of a PU binder; the results showed that with the addition of PU, the water absorption capacity of PCM (Portland cement mortar) and RHPC mortar, and RHP (rapid hardening Portland cement mortar) decreased by 67.97% and 23.46%, respectively.

In recent years, a series of scientific explorations have been conducted on the mechanical properties and damage modes of grouting concrete stones. Rentai et al. [24] investigated the mechanical properties of the grouted reinforcement of a fractured rock mass and validated their model via an indoor uniaxial compressive test. Shuai et al. [25] explored the molding effect of grouted body specimens and their damage modes under different pressure conditions through theoretical analysis, experimental study, and data generalization. Ma et al. [26] conducted grouting reconstruction experiments on fractured rock bodies in the laboratory to simulate the effect of grouting reinforcement. Liu et al. [27] conducted systematic tensile property tests on grouted and reinforced rock cracks and reported an increase in the tensile strength of the cracks with increased grouting viscosity. Zang et al. [28] revealed the deformation and damage mode of a grouted reinforcement and elaborated on the damage mechanism of the grouted reinforcement with different grain sizes. Zhang et al. [29] used theoretical analyses, physical experiments, and field tests to investigate the mechanism of the self-stressing grouting reinforcement of fractured rocks and its reinforcement effect.

In addition, in grouting pumping, according to the pressure provided by the equipment, it can be divided into static pressure grouting and high-pressure grouting. The high-pressure grouting is transported by high-pressure pump and a jet flow with huge energy is generated through the high-pressure nozzle. Therefore, the high-pressure grouting is more suitable for soil grouting, while the static pressure grouting is more suitable for the tunnel rock grouting condition discussed in this study. The working conditions studied in this paper are mainly tunnel fracture conditions, which often use static pressure grouting to pump grout. Grouting pumping is usually divided into single-liquid grouting and double-liquid grouting, and the double-liquid grouting pumping technology is usually used in the cement–water glass double-liquid reaction grouting pumping project. Its working principle is that the cement and water glass react to quickly block and realize the plugging and repair of the project. In this paper, the main focus is on the single-liquid grouting technology. The modified grouting material is developed by article research, and the solidification time can be controlled in about 100–500 min, which is suitable for pipeline pumping of ordinary grouting equipment.

The above research results indicate that comprehensive polyurethane-modified cement grouting material utilization is now garnering extensive attention at home and abroad. At present, an extensive body of research literature mainly focuses on the static mechanical properties of polymer grouting materials. While the mechanical properties of polymer grouting materials with dynamic load are also of great significance to their application and development, researchers have also carried out research on the dynamic mechanical properties. The study aims to provide ideas for polyurethane grouting repair materials for the shortage of water reaction materials in emergency rescue and advancing the comprehensive utilization of grouting materials.

In this study, a three-factor, four-level orthogonal test was conducted on WPU, hydroxypropyl methylcellulose (HPMC), and a WRA with different dosages to determine the optimal mix ratio of WPU-modified cement grouting materials. Based on the optimal mix ratio, the durability and micromechanism of the modified materials were investigated under the action of sodium sulfate freezing and thawing. In addition to this, specimens of SHPB’s grouting concretion stones were prepared using the slurry of the grouting material with the optimal mixing ratio, and the dynamic mechanical properties and energy characteristics of the grouting concretion stones were analyzed.

This paper studies the reinforcement treatment of water-rich fractured rock mass by waterborne polyurethane grouting materials, which can not only effectively deal with the risk of groundwater, but also improve the stability of stone body and the ability to resist dynamic load after grouting, so as to protect the safety of personnel and property and ensure the smooth progress of the project. It has important research background and application significance for promoting the safety and sustainability of underground engineering construction.

## 2. Materials and Methods

### 2.1. Test Materials

#### 2.1.1. Waterborne Polyurethane (WPU)

WPU can be classified into one- and two-component WPU. The PU used in this study, which is one-component WPU, was produced by Guangzhou Kedun Waterproof Material Co., Ltd. (Kedun Co., Guangzhou, China); it can be directly used for water plugging. The slurry is a brownish-yellow liquid, viscous, with a pungent odor, and is nontoxic after reaction with water. The specific factory parameters are shown in Table 1.

#### 2.1.2. Cement

**Figure 1:**
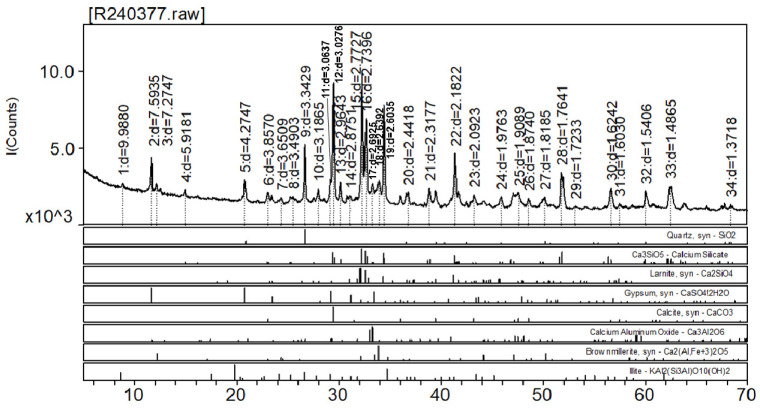
Mineral composition of cement.

Ordinary Portland Cement produced by Hailuo Co. Shandong province China (Hailuo Co., Binzhou, China) was used in this study. Mineral composition in Table 2 analyzed by XRD shows C_3_S is the main mineral. Figure 1 shows the specific composition. Its main physical parameters are shown in Table 3.

#### 2.1.3. Hydroxypropyl Methyl Cellulose (HPMC)

**Figure 2:**
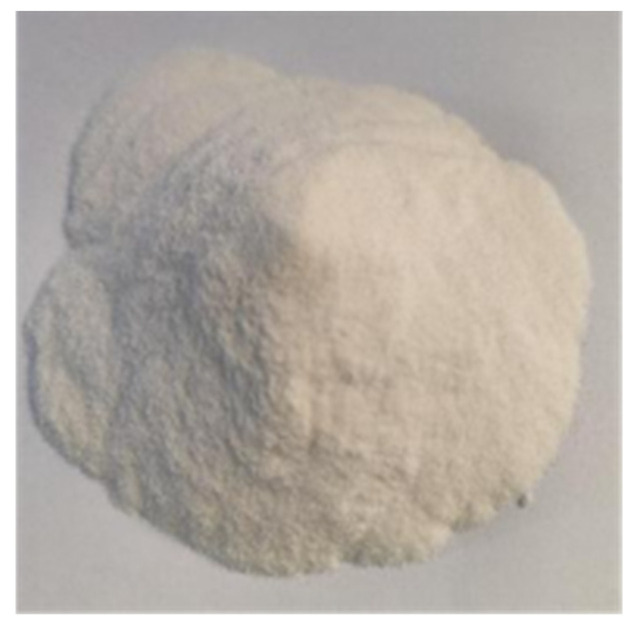
Hydroxypropyl methyl cellulose.

The HPMC used in the test is shown in Figure 2, and was produced by Jinzhou City Shunyue Building Materials Technology Co (Jinzhou, China). It is a nontoxic white powder with a viscosity of 25 w; it can be dissolved in cold water to form a transparent viscous solution. Hydroxypropyl methyl cellulose (HPMC) is a common admixture in factory made mortars. The methoxyl content appears as the key parameter of the hydration delay mechanism. The parameters of hydroxypropyl methyl cellulose are shown in Table 4.

#### 2.1.4. Water-Reducing Agent (WRA)

Water-reducing agents are an essential admixture that serves two crucial functions in concrete mixing. Firstly, they can reduce the amount of water required for the mixture while still preserving the desired fluidity of the fresh concrete. This enables the production of concrete with lowered water–cement ratios, ultimately enhancing its strength and durability. Secondly, water-reducing agents can augment the fluidity of fresh concrete. Therefore, WRA is capable of producing great water reduction, great flowability, or both, without causing undue set retardation or air entrainment in cementitious paste. The WRA used in the test was a naphthalene WRA provided by Shandong Wanshan Chemical Co (Wanshan Co., Weifang, China). It conforms to the national Method for Testing Uniformity of Concrete Admixtures.

### 2.2. Specimen Preparation

The specimen preparation process was divided into two parts: (1) the preparation of WPU-modified cementitious grouting materials and (2) the preparation of the grouting concretion stone according to the optimal ratio.

#### 2.2.1. Preparation of WPU-Modified Grouting Materials

We employed the L_16_ (4^3^) orthogonal test method to determine the setting time, fluidity, flexural strength, and compressive strength of the WPU-modified cement grouting materials (hereinafter referred to as the modified grouting materials) to determine the optimal ratio. The three factors were WPU as a percentage of the cement mass, HPMC as a percentage of the cement mass, and WRA as a percentage of the cement mass, denoted as factors A, B, and C, respectively. The four levels under each factor are shown in Table 5. This test was conducted at a fixed water–cement ratio of 0.5.

A total of 17 groups of mixing ratios were designed for the test—a control group (pure cement slurry with a water–cement ratio of 0.5) and the modified grouting materials prepared as 40 × 40 × 160 mm^3^ specimens. Based on the age as the object of study, the compressive strengths were determined at 3, 7, and 28 d; thus, three specimens were prepared for each group. In total, 153 specimens were prepared.

#### 2.2.2. Preparation of Grouting Concrete Stone

**Figure 3:**
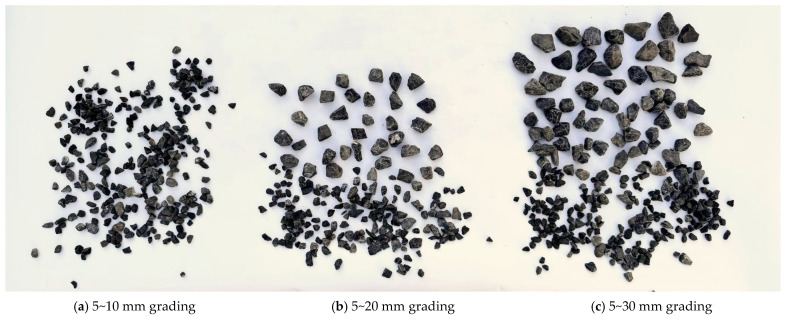
Continuously graded basalt stones.

In this study, basalt crushed stone obtained from Jinzhou City, Liaoning Province was used for the nodular body aggregate, and three grain size grades of 5–10, 5–20, and 5–30 mm were selected (Figure 3). The grading requirements for fine aggregates under ASTM C33 [30] dictate that no more than 45% should pass through a single sieve, with the remainder being retained on the next consecutive sieve. The quality test was performed in accordance with JGJ52-2006 Standard [31] for Technical Requirements and Test Method of Sand and Crushed Stone (or Gravel) for Ordinary Concrete. To quantify the effect of different grain size distributions on the amount of crushed stone, Φ100 × 50 mm^2^ grinding tools were used to lay various grain sizes of crushed stones to ensure surface smoothness and we calculated the tare weights of the grinding tools filled with crushed stones; we repeated these three times for each grain size distribution and used the average as this final result. The results show that the gravel weights for 5–10, 5–20, and 5–30 mm grain size distributions are 352, 343, and 333 g, respectively.

**Figure 4:**
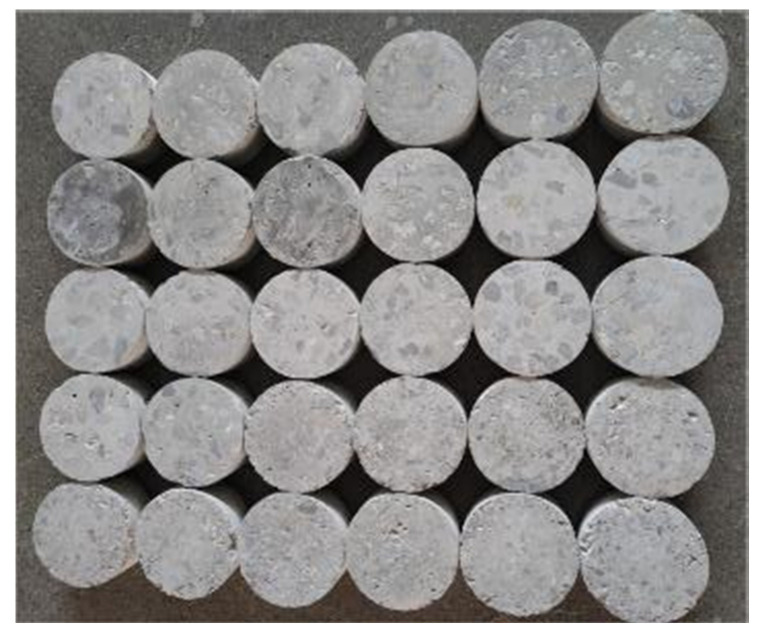
Smoothing part of the specimen.

According to the optimal ratio obtained from the experimental study, which was shown both in Table 5 and Table 6, a slurry was prepared by adding 0.05% HPMC, 5% WPU, and 0.2% WRA. The slurry was slowly poured along one side of a cylinder to aid the removal of air bubbles and subsequently vibrated. The demolding operation was performed after 48 h of resting. The cured specimens were then smoothed (Figure 4).

**Figure 5:**
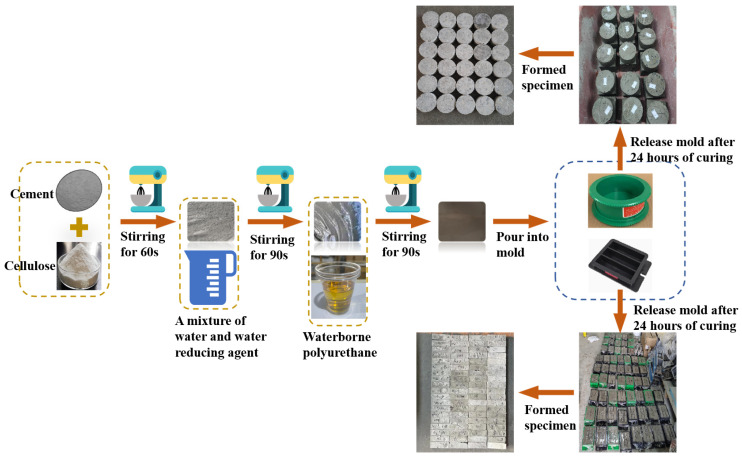
Specimen preparation process and experimental operation procedure.

In the three-factor, four-level orthogonal test and the durability test, all the specimens were of the same size, 40 × 40 × 160 mm^3^. Meanwhile, for the impact resistance test of grouting concretion stones, cylindrical specimens of size Φ100 mm × H50 mm were used. The specific experimental operating procedure is shown in Figure 5.

### 2.3. Test Methods

#### 2.3.1. Setting Time Test Method

**Figure 6:**
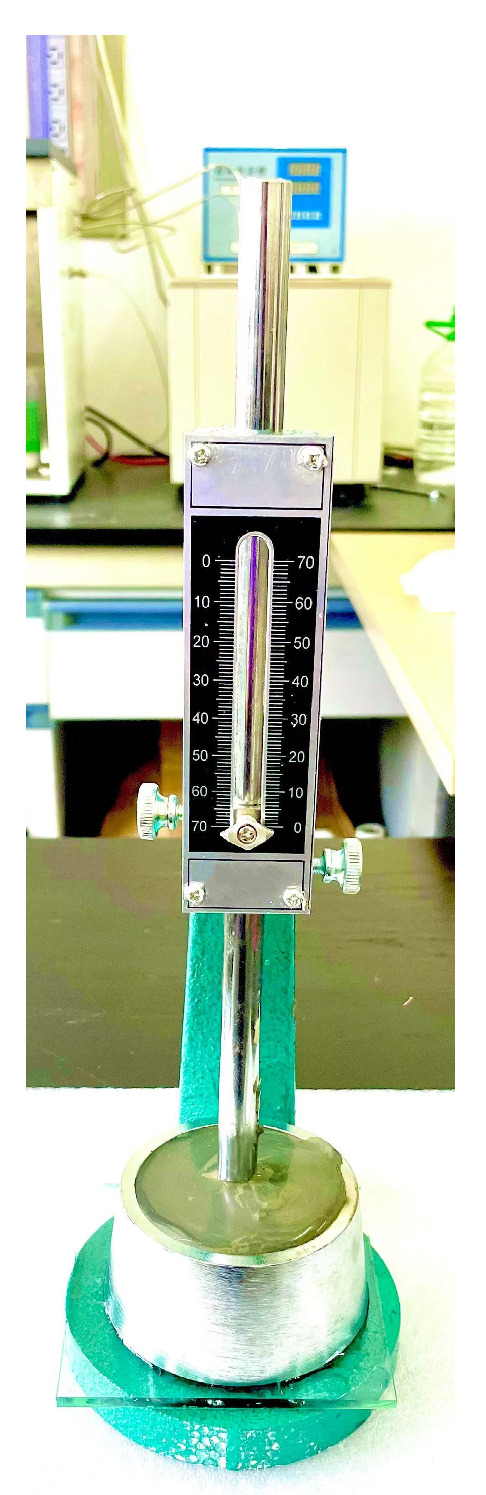
Vicat apparatus.

The setting time test is the core test to check the performance of the modified grouting materials. Some materials contain accelerating admixtures to reduce the setting time. This is particularly true of repair mortars and other spray-applied materials, so that strength gain can be fairly rapid. It is important to keep moving when using these types of materials. Once the material is mixed, it must be pumped immediately and kept in motion, and subsequent batches must be mixed and pumped as rapidly as possible. It is conducted in accordance with the provisions of Test Methods for Water Requirement of normal Consistency, Setting Time and Soundness of the Portland Cement, which is referable with ASTM C191-21 [32] Standard Test Methods for Time of Setting of Hydraulic Cement by Vicat Needle. A Vicat apparatus produced by Wuxi province China Zhongke Co (Wuxi, China). Was used in this test, which maximum sliding stroke is 70 mm (Figure 6).

First of all, the determination of the initial setting time: the specimen is placed in a humid environment after adding water for 30 min for the first measurement. At this time, the circular mold is removed from the self-humidifying air box and placed under the measuring needle. After lightly touching the slurry level of the modified grouting material, it is quickly loosened so that the measuring needle can fall vertically and freely. The observation needle stops falling within 30 s after release, when the distance from the needle to the bottom plate is 4 mm ± 1 mm, marking that the cement slurry has reached the initial setting stage, and the time required from adding water to reach this state is the initial setting time, expressed in min. Secondly, the determination of the final setting time: in order to accurately record the penetration of the needle, the measuring needle is equipped with a ring attachment. After the initial setting time is determined, immediately translate the test mold and slurry from the glass plate and turn it over 180°, ensure that the big end is facing up and the small end is facing down, and then put it back into the wet air box for further maintenance. When the final setting is close, the test is performed every 15 min, when the depth of the measuring needle is only 0.5 mm, and the ring attachment cannot leave a mark on the test, marking the cement slurry has reached the final setting stage. The time required from mixing cement with water to reach this state is called the final setting time and is expressed in min.

#### 2.3.2. Fluidity Test

**Figure 7:**
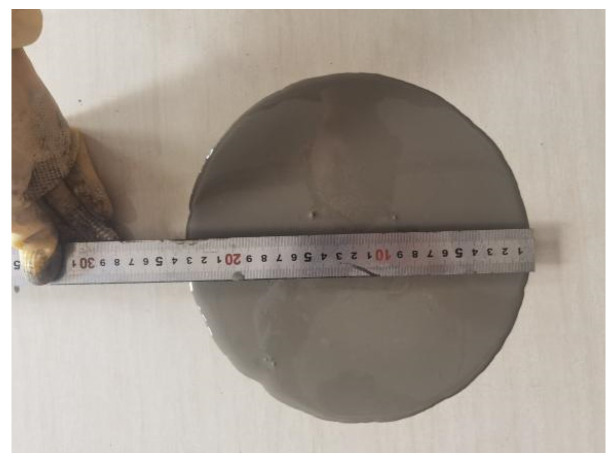
Net slurry flow test film.

Behavior in the pumping of grouting materials depends on the material’s consistency and rheological properties. Most materials need to be flowable or pourable for successful pumping. As shown in Figure 7, To ensure the setting time controllability, the flow of the modified grouting material is a key index to assess performance. Flowability was determined using the method stipulated in the ASTM C1437-20 [33] Standard Test Method for Flow of Hydraulic Cement Mortar. Specifically, the flowability of the grouting material was tested using a cement net slurry flowability test mold of a specific size, 36 × 60 × 60 mm^3^, which is produced by Hebei Jingrui Company of China. The glass plate used measured 400 × 400 × 5 mm^3^ and was supplemented with a steel straightedge. First, the glass plate was placed on a level work surface, and the flow mold was thoroughly cleaned, as well as the glass plate, agitator, and stirring pan with a damp cloth to ensure that all surfaces are free of water traces. Then, the cement slurry flow grinding tool was positioned in the center of the glass plate and covered with a wet cloth to keep it moist for ready use. Pour the pre-mixed slurry into the flow test mold, and immediately smooth the surface of the slurry with a scraper. Then, lift the flow test mold upright, and when the slurry stops flowing (more than 30 s), use a steel ruler to measure the maximum diameter of the slurry diffusion along two vertical directions, and use the larger of the two measured values as the final slurry flow index.

#### 2.3.3. Compressive Strength Test

**Figure 8:**
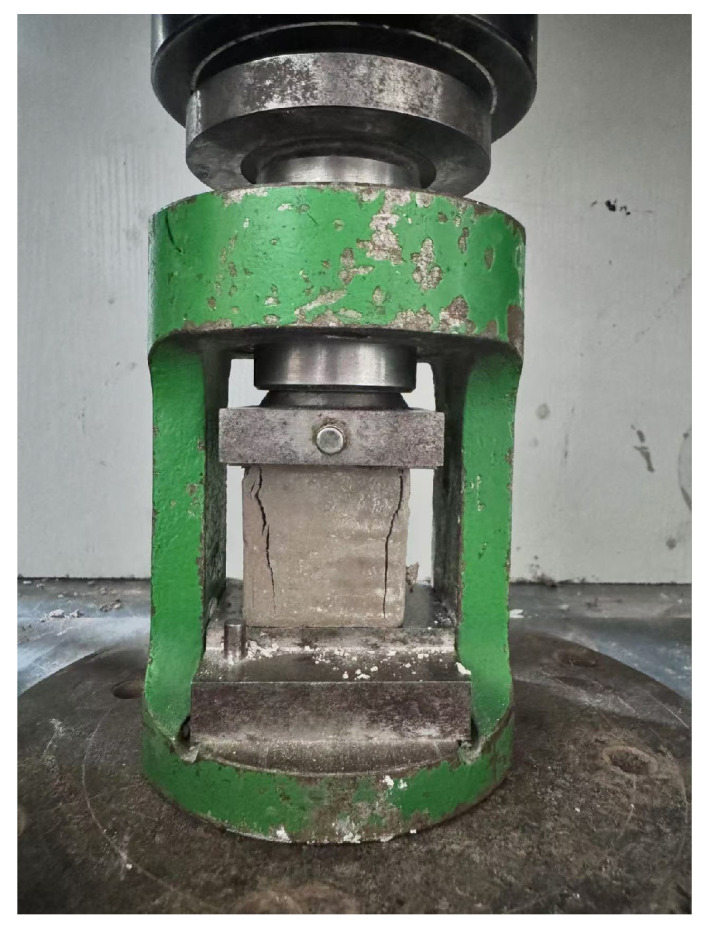
Compressive strength test.

In accordance with the provisions of the Test Method for Cement mortar Strength and the Test Code for Polymer Modified Cement Mortar, as shown in Figure 8, the specimens were prepared for a compressive strength test. The area of the stressed surface is 40 × 40 mm^2^. The WDW-4206 electro-hydraulic servo control test machine, which is produced by Hebei province China Kexi Co. (Shijiazhuang, China), was used in this test; the distance between two fulcrums is set to 100 mm to carry out the strength test. The loading rate was maintained at 0.2 mm/s until the specimen was broken, and the load value was recorded at this time. At least 3 specimens were prepared for each bending test, and we took the average value of the three tests with an accuracy of 0.01 MPa.

#### 2.3.4. SHPB

**Figure 9:**
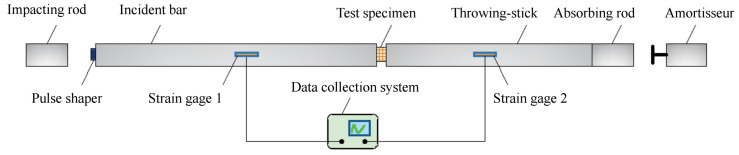
Schematic diagram of the SHPB unit.

The test equipment was manufactured by Qinhuangdao Longke Measurement and Control Technology Co (Qinhuangdao, China). A schematic of an SHPB device is shown in Figure 9. We used an SHPB device with a 100 mm diameter provided by the laboratory of Shenyang University of Technology. The device has a modulus of elasticity of 210 GPa and a density of 7850 kg/m^3^. The propagation equation of the stress wave in the rod is $C=\sqrt{E/R}$, with a wave speed of 5172.2 m/s.

**Figure 10:**
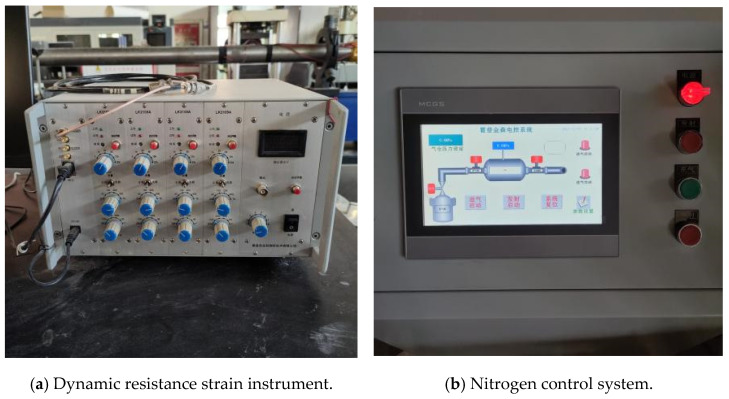

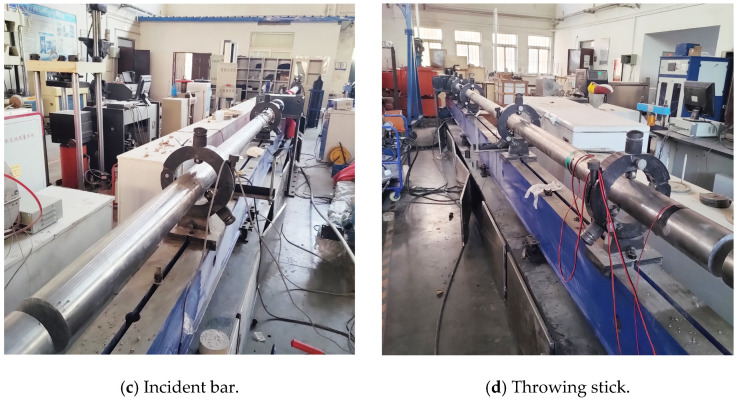
Physical diagram of the SHPB test setup.

A physical diagram of the SHPB test setup is shown in Figure 10. The polished grating concrete stone specimens were subjected to SHPB impact tests at three different impact pressures, i.e., 0.2, 0.3, and 0.4 MPa. Three experiments were performed under each test condition, and the results were averaged. Before the test, the grouting concretion stones were numbered and grouped into groups A, B, and C. Group A contains 5–10 mm continuous graded grouting concretion stones, denoted as 0.2-A, 0.3-A, and 0.4-A based on the corresponding impact air pressure. Group B contains 5–20 mm continuous graded grouting concretion stones, denoted as 0.2-B, 0.3-B, and 0.4-B based on the corresponding impact air pressure. Group C contains 5–30 mm continuous graded grouting concretion stones, denoted as the 0.2-C, 0.3-C, and 0.4-C based on the corresponding impact air pressure. Hereinafter, they are collectively referred to as the A, B, and C grouting concretion stones, respectively. The test program is summarized in Table 7.

## 3. Results and Discussion

### 3.1. Influence of Different Factors on the Initial and Final Setting Times and Fluidity

**Figure 11:**
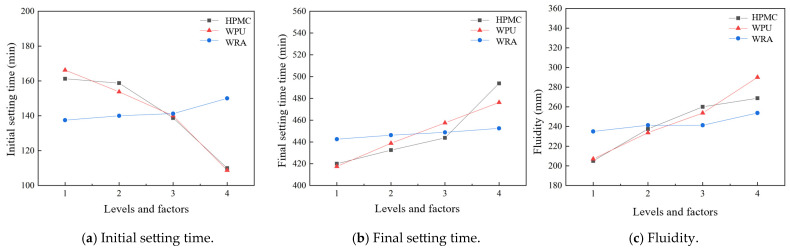
Effect of different factors on initial and final setting time and fluidity.

The effects of various level factors on the initial setting time, final setting time, and fluidity are summarized in Table 5 and Table 6. The experimental results are shown in Figure 11.

From Figure 11a, the initial setting time of the WPU-modified grouting material decreases with an increase in the HPMC and WPU dosages but increases with an increase in the WRA dosage. An increase in HPMC and WPU facilitates the hydration reaction, while an increase in WRA improves the fluidity but somewhat affects the hydration reaction rate, resulting in a prolonged initial setting time [34]. From Figure 11b, the final setting time of the WPU-modified grouting material was gradually prolonged with an increase in the HPMC, WPU, and WRA dosages. HPMC increases the viscosity and stability of the modified grouting material slurry and slows down the hydration reaction. When the dosage of HPMC, WPU, and the WRA is increased at the same time, their combined effects lead to enhanced hydration reaction in the slurry of the grouting material in the early stage; however, in the later stage, due to the combined effects of the restricted movement of water molecules, obstruction of diffusion of the hydration reactants, and reduction in the effective molecular weight of water, the hydration reaction rate reduces, leading to a prolonged final setting time. From Figure 11c, with an increase in the HPMC, WPU, and WRA dosages, the fluidity of the WPU-modified cementitious grouting material gradually increases. The increase in the WPU and WRA dosages promotes the dispersion and wettability of the cement particles and other solid components, reduces the cohesion of the mixture, and maintains the water retention level. This combined effect increases the fluidity of the overall mixture. Therefore, a simultaneous increment in the three combined components improves the fluidity and contributes to the grouting material workability.

### 3.2. Effect of Different Factors on Compressive Strength

**Figure 12:**
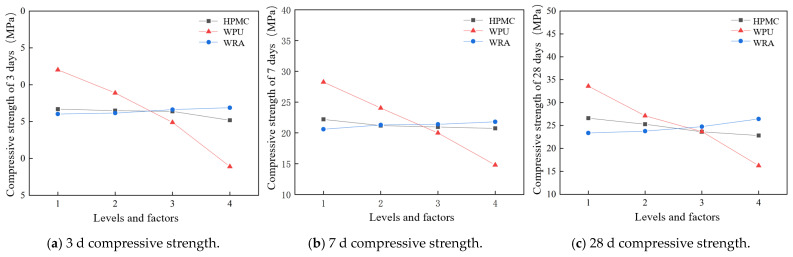
Effect of different factors on compressive strength at different ages.

From the analysis of the effect of each factor on the compressive strength in Figure 12, an increase in the WPU, HPMC, and WRA dosages gradually decreased the 3-d, 7-d, and 28-d compressive strengths. Although WPU is beneficial in improving early water resistance and adhesion, it may simultaneously hinder the further progress of the hydration reaction, thereby affecting strength development. WPU improves the interfacial properties of the modified grouting materials; however, if too much WPU is added, it may lead to inhomogeneity of the internal structure, which affects the strength. HPMC can considerably increase the viscosity of the cement slurry and improve the initial stability. However, an excessive viscosity may limit the movement and rearrangement between the cement particles, which is unfavorable to the hydration reaction, thereby affecting the compactness and strength of the modified grouting materials. Moreover, if HPMC is doped too much, it may lead to excess water during the hydration reaction, which in turn affects the quality and strength of the products. Theoretically, WRA can improve the material density and strength by improving the fluidity of the cement slurry and reducing the water consumption. However, excessive WRA may lead to excessive dispersion of the cement particles, reduce the effective contact area between the cement particles, and inhibit the hydration reaction, thereby affecting the strength. An increase in the three factors mentioned above improves the setting time and fluidity of the grouting material at the initial stage but an excessive addition adversely affects the normal hydration reaction of cement, resulting in insufficient hydration and low-quality hydration products, which in turn affect the densification and strength of the modified grouting material.

### 3.3. Linear Regression Analysis of Compressive Strength

When the age of the WPU-modified grouting material was 28 d, the compressive strength results obtained were subjected to linear regression analysis; the results are summarized in Table 8. According to the data analysis results (Table 8), the adjusted R^2^ value of the linear regression model reached 0.959, which means that the model could explain 95% of the correlation of the experimental data, indicating a high fitting accuracy. Meanwhile, the value of the Durbin–Watson statistic was 1.659, which is close to the ideal value of 2, indicating that there is no autocorrelation between the data. This result effectively excludes the possibility of pseudo-regression, thereby proving the accuracy and reliability of the model.

From Table 9, the significance coefficients of WPU, HPMC, and WRA are 0.000, 0.001, and 0.022 respectively, which are <0.05. WPU and HPMC have a more significant effect on the 28-d compressive strength of grouting materials. By the coefficient B, the three factors are considered to more accurately express the linear regression equation:

(1) $$f_{cs}=43.751-21.680x_{1}-1.178x_{2}-10.178x_{3}$$

where $f_{cs}$ denotes the 28-d compressive strength and $x_{1}$, $x_{2}$, and $x_{3}$ denote the HPMC, WPU, and WRA dosages, respectively.

### 3.4. Results and Analysis of Grouting Concrete Stones

The received data were processed using the three-wave method to obtain the results of the dynamic impact tests on the grouting concrete stone (Table 10).

From Table 10, the peak stress of the grouting concretion stones under different continuous distributions increases with the impact air pressure. When the stone grain size is 5–10 mm, the strain rates obtained at 0.2, 0.3, and 0.4 MPa are 40.50, 66.79, and 88.80 s^−1^, respectively, and the corresponding dynamic compressive strengths are 32.71, 54.48, and 114.37 MPa, respectively. When the stone grain size is 5–20 mm, the strain rates obtained at 0.2, 0.3, and 0.4 MPa are 39.67, 60.27, and 77.56 s^−1^, and the corresponding dynamic compressive strengths are 25.96, 48.75, and 78.41 MPa, respectively. When the grain size distribution is 5–30 mm, the strain rates obtained at 0.2, 0.3, and 0.4 MPa are 35.85, 52.01, and 71.82 s^−1^, and the corresponding dynamic compressive strengths are 21.43, 39.22, and 65.00 MPa, respectively.

#### 3.4.1. Damage Characteristics of Grouting Concrete Stones Under Different Impact Air Pressures

**Figure 13:**
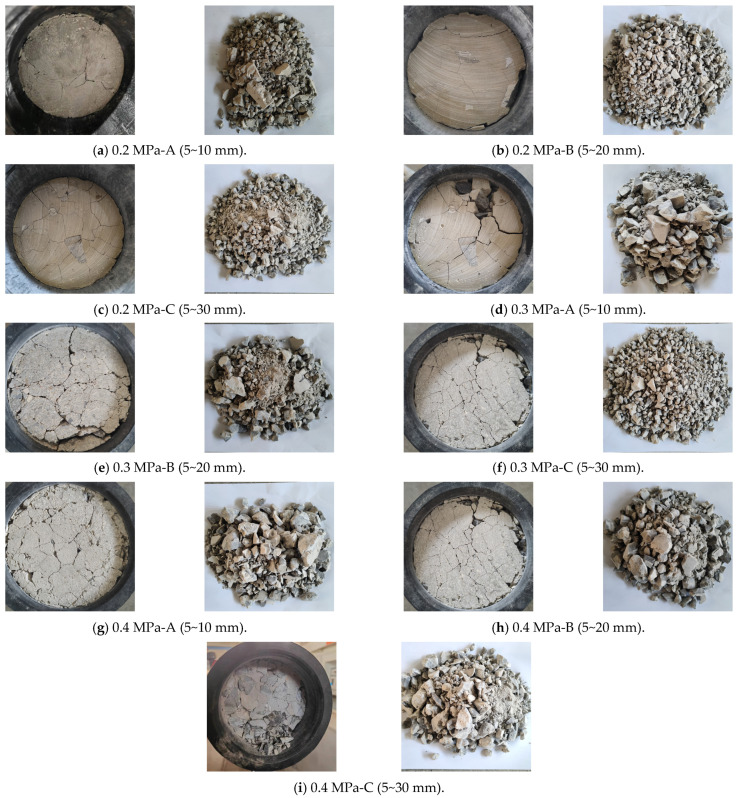
Damage characteristics of specimens under different impact air pressures.

After test completion, specimens were collected, organized, and photographed for each group at different impact air pressures; the crushing characteristic diagrams of the grouting concretion stones are shown in Figure 13.

Figure 13 demonstrates the damage characteristics of the specimens under different impact air pressure conditions. From the figure, the grouting material slurry can fully cover the stones under all working conditions, which clearly proves its excellent injectability. With an increase in the strain rate, the number of blocks after the grouting concrete stone destruction increases and the block size decreases. A comparison of the damage patterns of different stone grain sizes of the nodular bodies at the same strain rate reveals that an increase in stone gradation increases the number of broken blocks after the specimen impact. The reason behind this phenomenon is that the smaller the stone size, the denser the interior of the nodule, the smaller the number of internal microdefects, and the smaller the number of cracks under the impact load, which leads to a lighter degree of specimen damage.

#### 3.4.2. Stress–Strain Curve Analysis of Grouting Concrete Stone Under Different Impact Air Pressures

**Figure 14:**
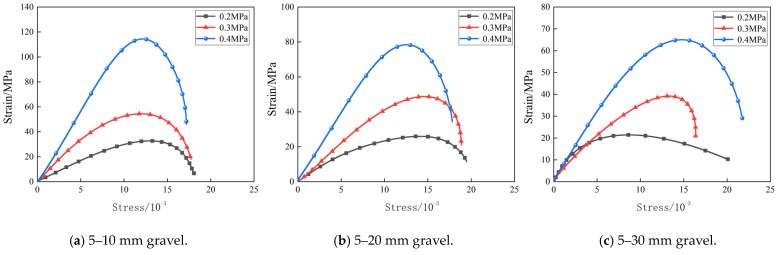
Strain–stress curves for different impact air pressures.

The dynamic stress–strain relation of the grouting concretion stones is crucial for understanding the dynamic properties of the grouted materials and analyzing the dynamic strengths, stress–strain, and other indexes under impact loading. Figure 14 shows the stress–strain curves of the grouting concrete stone specimens of 5–10, 5–20, and 5–30 mm continuous gradations.

As shown in Figure 14, the stress–strain curves under different impact air pressures are basically the same as the change in impact air pressure for the grouting concrete stone under the above three groups of continuous gradation. That is, with an increase in the impact velocity, the peak stress of the grouting concrete stone specimens gradually increases, showing an obvious strain rate effect. This is related to the crack propagation rate of the grouting concrete stones and the stress wave propagation and reflection. The slope of the straight-line portion of the stress–strain curve is the dynamic modulus of elasticity, which is a physical quantity that describes the stiffness characteristics of a material under dynamic loading. The dynamic modulus of elasticity of the nodular body significantly increases with increasing impact velocity, attributable to the microstructural adjustments within the materials at high impact speeds, which enable more efficient load transfer.

#### 3.4.3. Effect of Strain Rate on the Dynamic Compressive Strength of Grouting Concrete Stone

**Figure 15:**
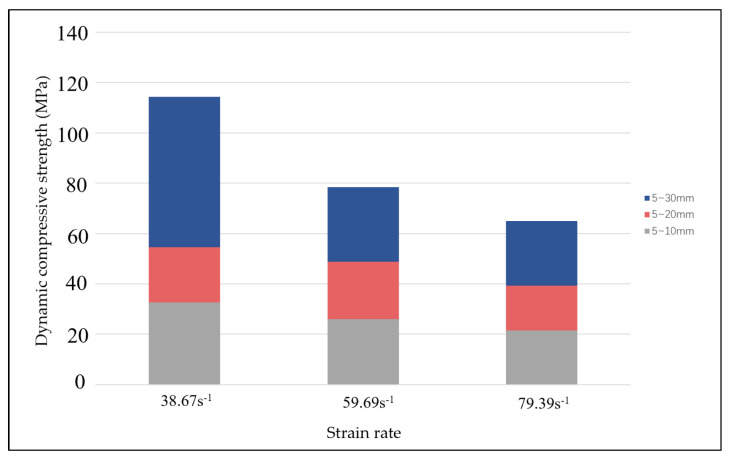
Effect of average strain rate on the dynamic compressive strength.

Figure 15 shows when the average strain rate is 38.67 s^−1^, the dynamic compressive strength of the A nodule body increased by 6.75 and 11.28 MPa compared with those of the B and C nodule bodies, respectively. When the average strain rate is 59.69 s^−1^, the dynamic compressive strength of the A nodule body increased by 5.73 and 15.26 MPa compared with those of the B and C nodule bodies, respectively. When the average strain rate is 79.39 s^−1^, the dynamic compressive strength of A was increased by 35.96 and 49.37 MPa compared to those of the B and C nodule bodies, respectively. This indicates that with an increase in strain rate, the larger the stone size, the more obvious the dynamic compressive strength decline. The reason for this is that under high strain rate conditions, the propagation rate of cracks in the materials may be limited because crack extension takes time to develop. When the strain rate is increased, crack propagation is inhibited, making the materials able to withstand greater stresses before damage occurs, thereby increasing their compressive strengths. At higher strain rates, the microstructure may produce stronger confinement effects and interaction forces in a short period, thereby enhancing the overall compressive properties of the materials.

**Figure 16:**
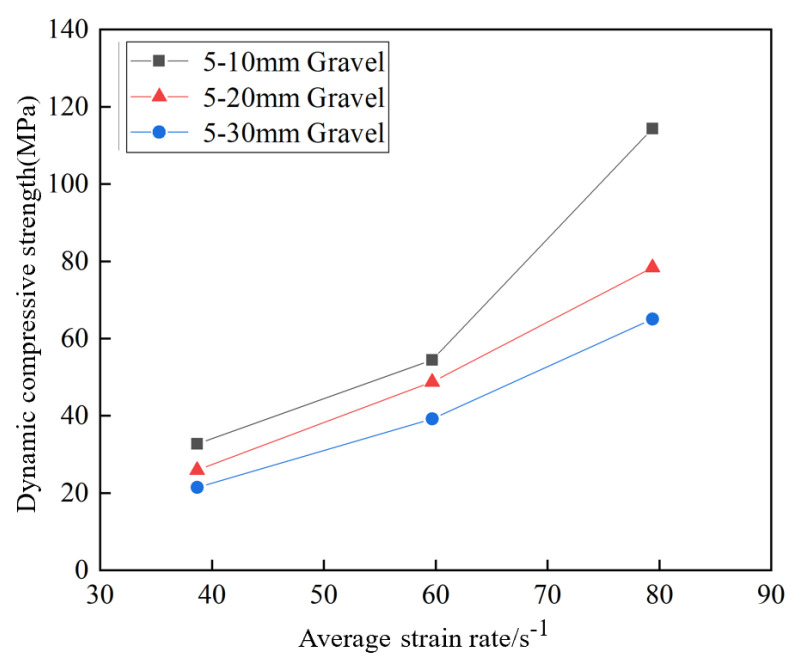
Effect of strain rate on the dynamic compressive strength of the grouting concretion stone.

Figure 16 shows the effect of the average strain rate on the dynamic compressive strength of the grouting concrete stones. From the figure, the dynamic compressive strength of the grouting concretion stones under the same grain size increases with the average strain rate. According to the above test data, the relation between the strain rate and dynamic compressive strength of the grouting concretion stones was analyzed as equation in Table 11.

#### 3.4.4. Effect of Different Stone Grain Sizes on the Dynamic Compressive Strength of Grouting Concrete Stone

**Figure 17:**
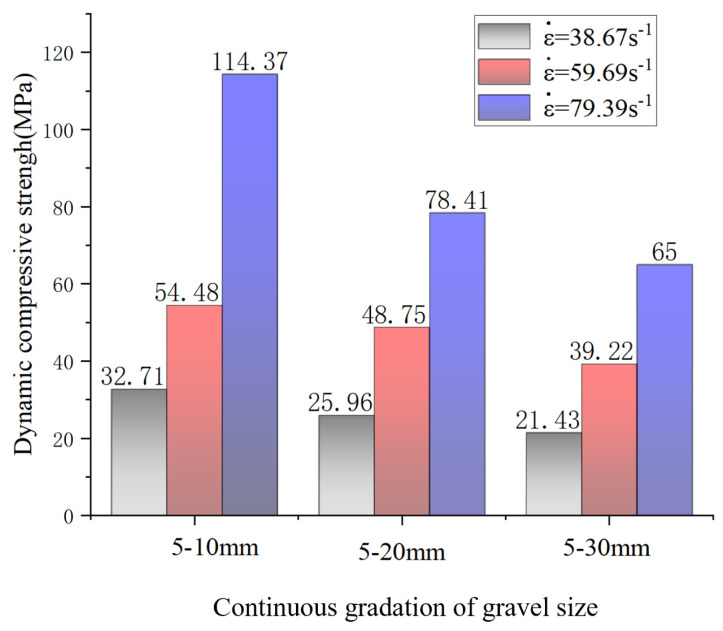
Effect of stone grain size on dynamic compressive strength of grouting concretion stone.

**Figure 18:**
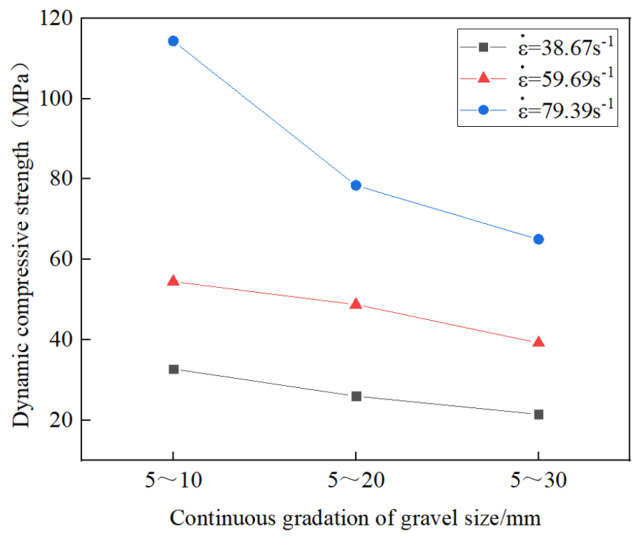
Effect of continuous graded average stone grain size versus dynamic compressive strength.

From Figure 17, the grain size of the continuously graded stones significantly affects the dynamic compressive strength. From the figure, it is found that the dynamic compressive strength of three groups of grouting concretion stones, A (5–10 mm), B (5–20 mm), and C (5–30 mm), decreases gradually with increasing stone grain size at the same average strain rate. The degree of influence of gravel size on the dynamic compressive strength follows the order d_5–10 mm_ > d_5–20 mm_ > d_5–30 mm_ (d denotes diameter). When the stone grain size is 5–10 mm, the dynamic compressive strength of the grouting concretion stones with an average strain rate of 79.39 s^−1^ is 59.89 and 81.66 MPa higher than those of the stones with average strain rates of 59.69 and 38.67 s^−1^, respectively. When the stone grain size is 5–20 mm, the dynamic compressive strength of the grouting concretion stones with an average strain rate of 79.39 s^−1^ is 29.66 and 52.45 MPa higher than those of the stones with average strain rates of 59.69 and 38.67 s^−1^, respectively. When the stone grain size is 5–30 mm, the dynamic compressive strength of the grouting concretion stones with an average strain rate of 79.39 s^−1^ is 25.78 and 43.57 MPa higher than those of the stones with average strain rates of 59.69 and 38.67 s^−1^, respectively. The main factor is an increase in stone size increases the porosity inside the materials. This is because larger stones have fewer contact points with each other for the same volume, resulting in more voids in the overall structure. These voids reduce the connectivity and tightness within the specimens, making the force transfer less uniform in larger-sized stones when subjected to impact, thereby reducing the dynamic compressive strength. According to the test data, the relation between the average stone grain size and dynamic compressive strength under continuous grading was shown (Figure 18).

### 3.5. Energy Characteristic Analysis of Grouting Concretion Stone

#### 3.5.1. Influence of Strain Rate on Energy Characteristics of Grouting Concretion Stone

To reflect the energy dissipation loss of the grouting concretion stones under different working conditions, the energy utilization rate *q* is calculated as follows:

(2) $$W_{I}=A_{0}CE\int_{0}^{t}\varepsilon_{I}^{2}\left(t\right)dt$$

(3) $$W_{R}=A_{0}CE\int_{0}^{t}\varepsilon_{R}^{2}\left(t\right)dt$$

(4) $$W_{T}=A_{0}CE\int_{0}^{t}\varepsilon_{T}^{2}\left(t\right)dt$$

(5) $$W_{D}=W_{I}-W_{R}-W_{T}$$

(6) $$q=\frac{W_{D}}{W_{I}},$$

where $W_{I}$, $W_{R}$, $W_{T}$, and $W_{D}$ denotes the incident, reflected, transmitted, and absorbed energies of the specimen under impact, respectively.

**Figure 19:**
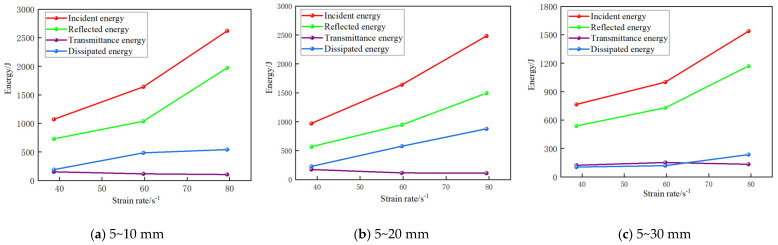
Effect of strain rate on the energy of grouted stone bodies for different stone grain sizes.

The results of calculating the energy under each condition using the above equation are shown below. Figure 19 shows the effect of the strain rate on the energy of the grouting concrete stones at different stone grain sizes. From the figure, the incident and reflected energies of the specimens with the same stone grain size show an increasing trend with an increase in strain rate. The transmitted energy shows different trends with an increase in strain rate. The dissipation energy increases with strain rate because the damage threshold of the grouting concretion stones and bearing capacity increase, and more energy is required for the grouting concretion stone destruction. The above analysis shows that the grouting concretion stones have better energy absorption and conversion ability under impact, which can effectively convert the external impact energy into the internal energy of the specimens while reducing the loss of energy through the form of crushing and emanation. By increasing the incident and reflected energies, the grouting concretion stones effectively control energy dissipation, which not only enhances their structural stability but also improves their performance under extreme working conditions.

**Figure 20:**
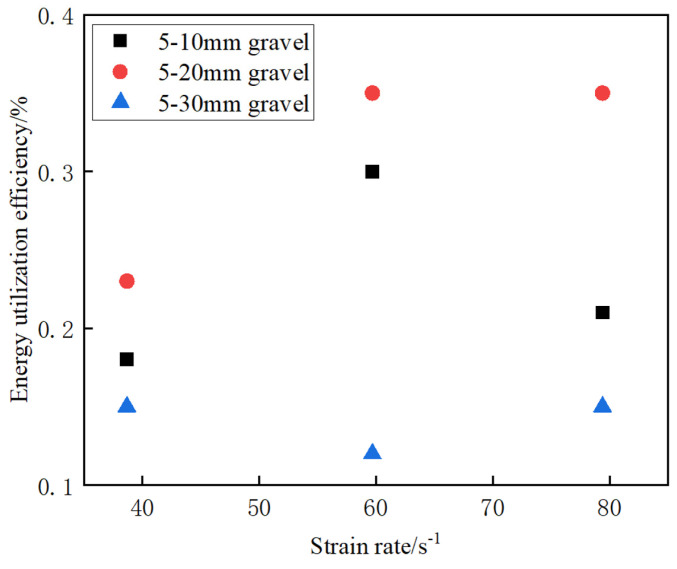
Effect of strain rate on energy utilization of grouting concretion stone for different stone grain sizes.

Figure 20 shows that the energy dissipation rate of the grouted nodular body exhibits a clear discrete relation with the strain rate. The energy dissipation rates of the grouted nodular bodies at different strain rates show specific independence, i.e., the energy dissipation rate neither increases nor decreases with an increase in the average strain rate. This may be because it is related to the internal structural characteristics of the grouted nodular bodies as well as the shapes of the constituent aggregates and their specific positions in the structure. The energy dissipation in the grouted stone bodies is not only affected by the change in the strain rate but also by the internal microstructures of the specimens. Internal specimen factors, such as the shape and size distributions of the aggregates, as well as the interaction between the aggregates, play a decisive role in the energy transfer and dissipation processes. Therefore, the relation between the average strain rate and energy dissipation is nonlinear, reflecting the far-reaching and complex influence of the internal structures of the specimens on their macroscopic mechanical response [35].

#### 3.5.2. Influence of Different Stone Grain Sizes on Energy Characteristics of Grouting Concretion Stone

**Figure 21:**
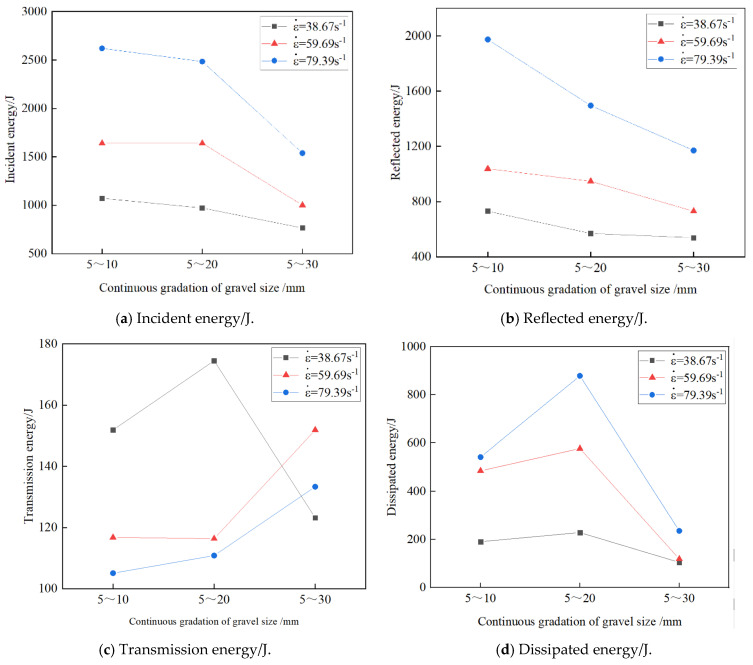
Effect of different stone grain sizes on the energy of grouting concretion stone.

Figure 21 shows the effect of different stone grain sizes on the energy of the grouting concrete stones. As shown in the figure, the dissipated energy first increases and then decreases with increasing stone grain size at the same average strain rate. This may be because a larger stone grain size may lead to a looser internal specimen structure, thereby increasing the internal voids. This structural change causes more energy to be used to overcome the resistance caused by the internal voids when the specimen is subjected to an external force, thereby increasing the energy dissipation. At larger grain sizes, cracks may need to bypass stones or pass through more interfaces, which increases the complexity and difficulty of crack propagation, thereby increasing the energy dissipation. As the grain size increases, the energy required for crack propagation increases, leading to an increase in the dissipated energy. In the specimens, an increase in the stone grain size may change the stress wave propagation characteristics. Larger stones may cause more scattering and attenuation of the stress wave during propagation, thereby reducing the amount of energy that reaches the other sides of the specimens (i.e., the transmitted energy) and the amount of energy that is reflected to the incident sides (i.e., the reflected energy). Thus, the incident and reflected energies decrease with increasing stone grain size.

**Figure 22:**
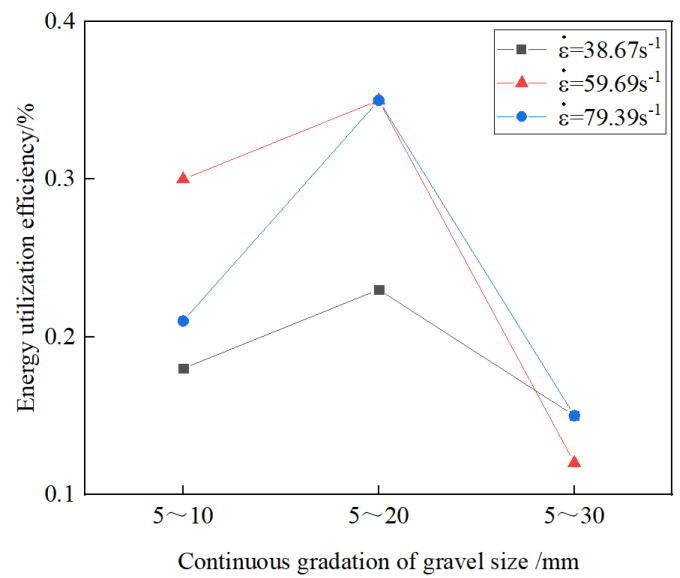
Effect of different stone grain sizes on the energy utilization of grouting concretion stone.

Figure 22 shows the effect of different stone grain sizes on the energy utilization of the grouting concrete stones. From the figure, at the same average strain rate, the energy utilization of the grouting concretion stones first increases and then decreases with increasing stone grain size. The reason is that a moderate increase in the stone size may improve the microstructure of the grouting concretion stones, such that the energy can be used more effectively to enhance the damage resistance of the grouting concretion stones. However, when the particle size exceeds 5–20 mm, there may be instability in the internal material structure and enhanced ineffective energy dissipation, thereby decreasing the overall energy utilization [36].

## 4. Conclusions

Herein, a series of laboratory tests were performed to evaluate the effectiveness of adding WPU, HPMC, and WRA to improve the properties of grouting materials in terms of the solidification time, compressive strength, and stone energy absorption. The following conclusions were drawn:

(1) Experimental studies were conducted on the durability of the modified cement grouting materials and the dynamic mechanical properties of the grouting concrete stones. By analyzing the working performance and mechanical properties, the optimal mixing ratio of the modified cement grouting materials was determined as follows: water–cement ratio of 0.5, HPMC content of 0.05%, WPU content of 5%, and WRA content of 0.2%. The modified cement grouting materials were prepared using WPU, cement, and cellulose. Via orthogonal test analysis, WPU had the most significant effect on the initial setting time, fluidity, and compressive strength and HPMC had the most significant effect on the final setting time.
(2) With increasing contents of HPMC and WPU, the initial setting time of the WPU-modified grouting materials was shortened. Further, with the increasing content of the WRA, the initial setting time increased. Finally, with increasing contents of WPU, HPMC, and the WRA, the 3-d, 7-d, and 28-d compressive strength gradually decreased.
(3) At curing for 28 d, regression analysis was performed on the compressive strength of the grouting materials and linear regression equations of the influence factors of the three external parameters (WPU, HPMC, WRA) were established. The significant coefficient of the WRA was 0.022, which showed the most significant effect on the 28-d compressive strength.
(4) Under impact loading, the degree of influence of the stone particle size on the dynamic compressive strength of the grouting concrete stone showed the order of d_5–10 mm_ > d_5–20 mm_ > d_5–30 mm_. The stone particle size with the highest influence was 5–10 mm, and the dynamic compressive strength of the grouting stone body at an average strain rate of 79.39 s^−1^ was 59.89 MPa and 81.66 MPa higher than those at average strain rates of 59.69 and 38.67 s^−1^. Under the same strain rate, the dynamic compressive strength decreased with increasing stone particle size. As the strain rate increased, the effect of the stone particle size on the dynamic compressive strength increased. Analyze by selecting an appropriate stone particle size, and the absorption energy and energy utilization rate of the grouting concretion stone are the highest when the stone particle size is 5–20 mm.

This study provides concepts for improvements in the comprehensive properties and applications of grouting materials. Considering material generalizability, in future research work, we will focus on more extreme environments, such as acidic or alkaline environments, as well as freeze–thaw properties and aging resistance. In addition, we will consider the effect of high temperatures on the grouting process and develop new types of grouting equipment to validate and extend our findings. We will also study the permeability and porosity of the grouting materials in more depth to enhance their durability.

## Figures and Tables

**Table 1:** Waterborne polyurethane parameters.

No.	Category	Standard Request	Inspection Result	Judge
1	Appearance	Homogeneous liquid, no impurities, No layering	Homogeneous liquid, no impurities, No layering	qualified
2	Density, g/m^3^	≥1.0	1.112	qualified
3	Viscosity, Mpa·s	≤1.0 × 10^3^	7.6 × 10^2^	qualified
4	Setting time, s	≤150	54	qualified
5	Expansion efficiency with water, %	≥20	77	qualified
6	Water contained (10 times water), s	≤200	86	qualified
7	No volatile solids content, %	≥75	81	qualified
8	Foaming rate, %	≥350	552	qualified

**Table 2:** Mineral composition of cement.

Specimen [R240377]	Mineral mass fraction × 10^−2^							
	C_3_S	C_2_S	CFA	CA_3_Al	Quartz	Gypsum	Illite	Calcite
Cement	52.4	11.6	5.5	2.0	6.3	6.6	1.6	14.0

**Table 3:** Physical parameters of cement.

Stability	Specific Surface Area/ m^2^/kg	Solidification Time/min		Compressive Strength/MPa		Flexural Strength/MPa	
		Incipient Condensation Time	Final Setting Time	3 d	28 d	3 d	28 d
Qualified	380	194	392	23.7	48.4	5.6	7.43

**Table 4:** Parameters of hydroxypropyl methyl cellulose.

Index Name	Analysis Result
Character	white powder
Moisture (%)	3%
Viscosity (Mpa·s)	250,000
Ash content (%)	3%
Light transmittance (%)	96%
Water-insoluble (%)	0.3%
Methoxy content (%)	29%
Gelation temperature (%)	65 °C
Hydroxypropyl content (%)	13%
PH	6.5

**Table 5:** Orthogonal test factor level table.

Levels	Factors		
	A (HPMC)/%	B (WPU)/%	C (WRA)/%
1	0.05	5	0.05
2	0.1	10	0.1
3	0.15	15	0.15
4	0.20	20	0.2

**Table 6:** Extreme difference analysis of 28 d compressive strength under different factors.

	A	B	C
	HPMC	WPU	WRA
*K*_1_	26.59	33.59	23.37
*K*_2_	25.27	27.11	23.76
*K*_3_	23.63	23.67	24.75
*K*_4_	22.81	16.25	26.42
*R*	3.78	17.34	3.06
The priority relationship of factors	B > A > C		
Optimal level	A_1_	B_1_	C_4_
Optimal combination	A_1_B_1_C_4_		

**Table 7:** Test protocol.

Grouting Concretion Stone	Specimen Quantity of Different Impact Air Pressure		
	0.2 MPa	0.3 MPa	0.4 MPa
A	3	3	3
B	3	3	3
C	3	3	3

**Table 8:** Summary of models.

R	R^2^	Adjusted R^2^	Errors in Standardized Estimates	Durbin-Watson
0.979	0.959	0.948	1.58984	1.659

**Table 9:** Coefficients of regression equations.

Equation Model	Non-Standardized Coefficient		Standardized Coefficient	Significance
	B	Standard-Inaccuracy		
(constant)	43.571	1.549		0.000
HPMC	−21.680	7.164	−0.179	0.011
WPU	−1.178	0.072	−0.973	0.000
WRA	−10.178	3.887	−0.156	0.022

**Table 10:** SHPB dynamic impact compression test results.

Serial No.	Air Pressure/MPa	Impact Velocity m/s	Strain Rate /s^−1^	Peak Strain	Dynamic Compressive Strength/MPa
0.2-A	0.2	7.97	40.50	0.029	32.71
0.3-A	0.3	10.56	66.79	0.032	54.48
0.4-A	0.4	12.86	88.80	0.024	114.37
0.2-B	0.2	7.75	39.67	0.008	25.96
0.3-B	0.3	10.39	60.27	0.028	48.75
0.4-B	0.4	12.67	77.56	0.028	78.41
0.2-C	0.2	7.62	35.85	0.007	21.43
0.3-C	0.3	10.21	52.01	0.024	39.22
0.4-C	0.4	12.32	71.82	0.017	65.00

**Table 11:** Results of the strain rate to the dynamic compressive strength number of the grouted nodular body.

Average Gravel Size	Quadratic Polynomial Fitting Equation	$\mathrm{Correlation}\;\mathrm{Coefficient}\;R^{2}$
7.5	$R=49.793-0.536x+0.002x^{2}$	0.9966
12.5	$R=52.521+0.235x-0.005x^{2}$	0.9942
17.5	$R=236.523-4.157x+0.025x^{2}$	0.9982

## Data Availability

The original contributions presented in the study are included in the article, further inquiries can be directed to the corresponding author.
